# Supplementary material for: Who speaks next? Multi-party AI discussion leveraging the systematics of turn-taking in Murder Mystery games
Source: Front Artif Intell. 2025 Jun 17;8:1582287. doi: 10.3389/frai.2025.1582287 (PMC12209177; doi:10.3389/frai.2025.1582287)
Supplement: Supplementary file 1 [file Data_Sheet_1.pdf]

Supplementary Material

1 SUPPLEMENTARY TABLES AND FIGURES

1.1 Tables

Table S1. Classification of Utterances that Lead to Dialogue Breakdowns. (Higashinaka et al., 2022)

Classification		
	**Form**	**Content**
**Response**	Ignore question<br>Ignore request<br>Ignore suggestion<br>Ignore greeting	Ignore expectation
**Context**	Unclear intention of utterance<br>Topic-change error<br>Lack of information	Self-contradiction<br>Interlocutor Contradiction<br>Repetition

1.2 Figures

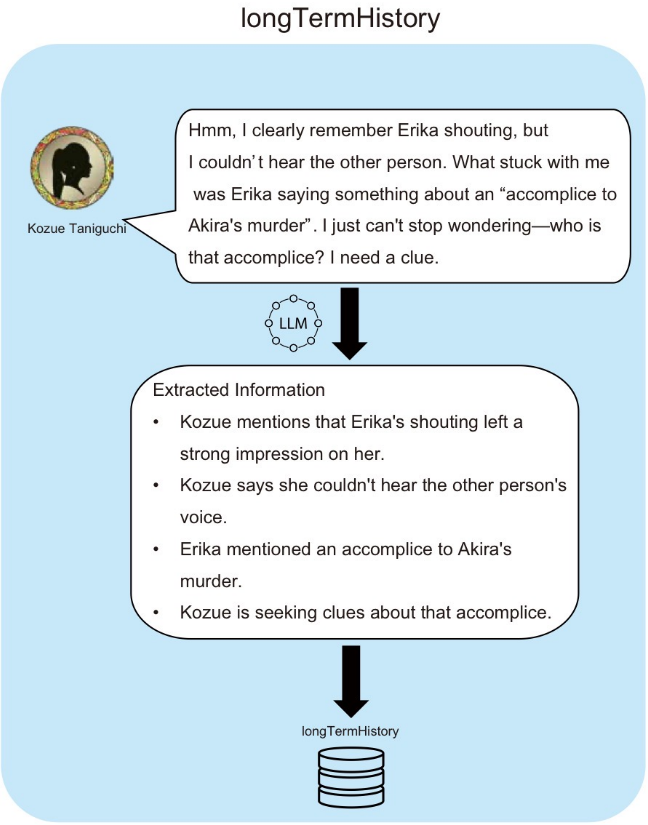

Figure S1. Example of normalizing utterances into knowledge or information and storing in longTermHistory.

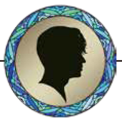

Character Information:
You are Masato Nishino.
${CharacterInfo}
...
Conversation history：
${History}
...
Your memory:
${shortTermHistory}
...
You can take the following Actions:

- Speak: Use when asking questions or giving explanations.
- Listen: Use when listening to someone's statement.

Formatting instructions:
Please use the following format for output:
Output:

```
{
    "Thought": "You should always think about what
    to do, but think concisely",
    "Action": "The action you should take. Either
    'Speak' or 'Listen'",
    "Importance": “The importance of that action (int: 0-9)”
}
```

Based on the information so far, decide what you should do next in the upcoming turn.
Now, let's begin. Trust yourself and push beyond your limits.
Generate concisely following the format:
Output:

Figure S2. The prompt for think().

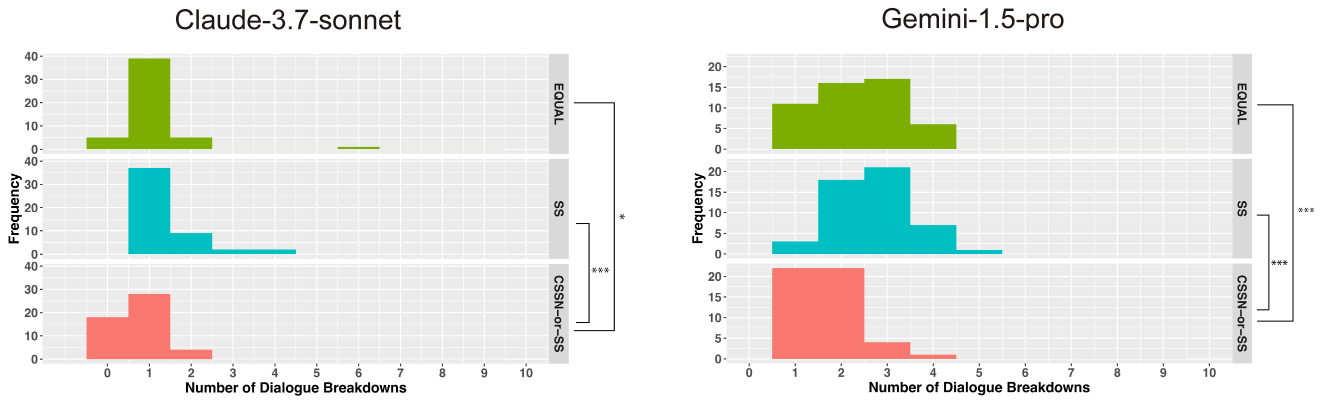

Figure S3. The results of the dialogue breakdown analysis conducted with Claude-3.7-sonnet and Gemini-1.5-pro.

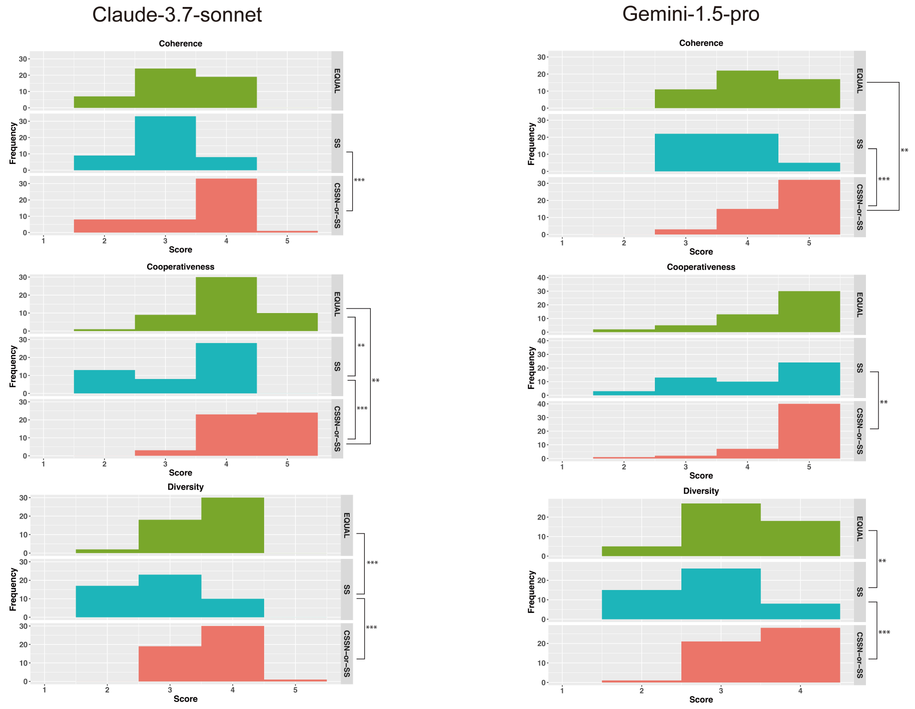

Figure S4. The results of the LLM-as-a-judge conducted with Claude-3.7-sonnet and Gemini-1.5-pro.

REFERENCES

Higashinaka, R., Araki, M., Tsukahara, H., and Mizukami, M. (2022). Classification of utterances that lead to dialogue breakdowns in chat-oriented dialogue systems. *Journal of Natural Language Processing* 29, 443–466. doi:10.5715/jnlp.29.443

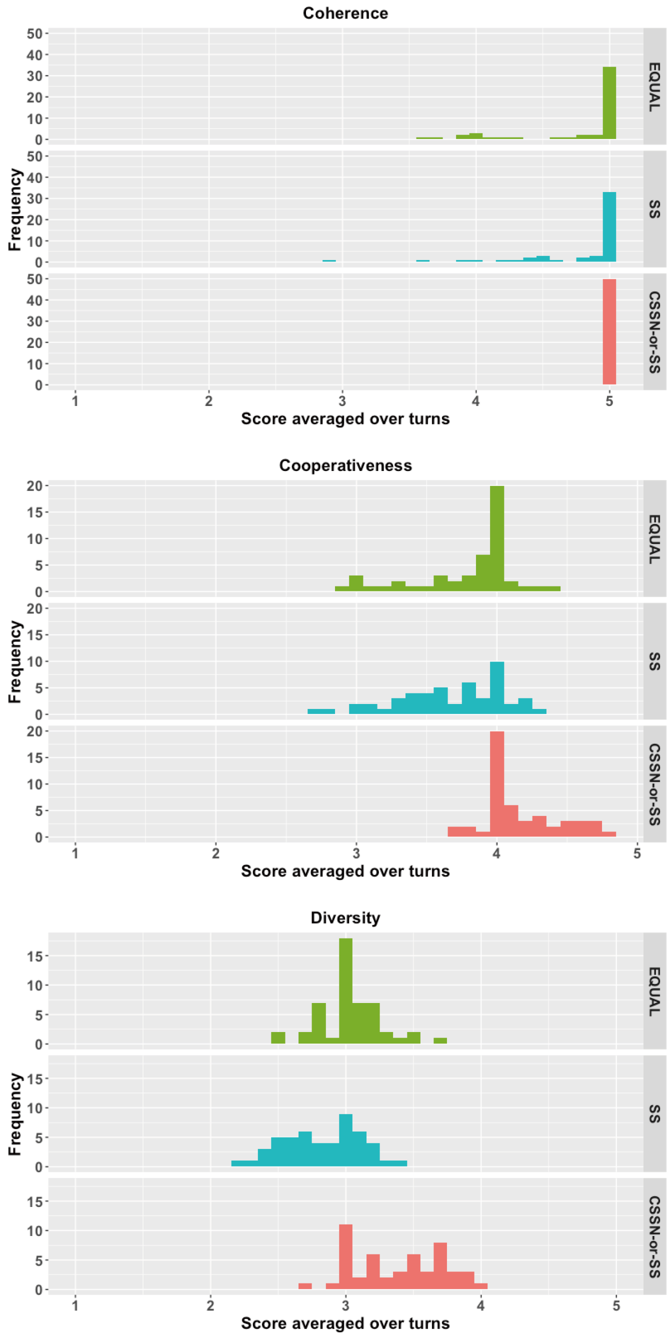

Figure S5. The results of LLM Judge on a per-turn basis.

1	Kozue:	"Um, everyone, about last night ... Around 21:45, I saw Erika arguing with someone near the observation deck. Does anyone know anything about this?"
2	Takeshi:	"Around 21:45 ... Ah, I was at the observation deck at that time too. But then I suddenly felt someone's presence and got scared, so I ran away."
3	Yukiko:	"Takeshi, when you ran away, didn't you see anyone?"
4	Takeshi:	"No, I felt someone's presence, but it was dark and I couldn't tell who it was."
5	Takeshi:	"Come to think of it, Yukiko, that bright red lipstick in your bag, do you use it?"
6	Yukiko:	"What, there was a bright red lipstick in my bag? I don't remember at all... Could it be someone's prank?"
7	Takeshi:	"It might be a prank. But I think that lipstick might be the one Erika had. Have any of you seen Erika wearing bright red lipstick?"
8	Kozue:	"Actually, I have seen Erika wearing bright red lipstick. But I wonder why that lipstick was in Yukiko's bag."
9	Masato:	"I wonder why that lipstick was in Yukiko's bag. Maybe it's related to Erika arguing with someone."
10	Masato:	"Yukiko, the fact that the lipstick was in your bag might mean someone put it there deliberately. Maybe Erika was trying to tell us something?"

Figure S6. Example of low score conversation.
